# Optimization of Sanitation Process Parameters of Slightly Acidic Electrolyzed Water for Automated Milk Feeders Using Response Surface Methodology

**DOI:** 10.3390/ani16081225

**Published:** 2026-04-16

**Authors:** Yunying Liu, Yu Zhang, Xinyi Du, Zhengxiang Shi, Chaoyuan Wang, Hao Li, Amingguli Yasheng

**Affiliations:** 1 College of Water Resources and Civil Engineering, China Agricultural University, Beijing 100083, China; liuyunying@cau.edu.cn (Y.L.); 2 Key Laboratory of Agricultural Engineering in Structure and Environment, Ministry of Agriculture and Rural Affairs, Beijing 100083, China; 3 Institute of Animal Husbandry and Veterinary Medicine, Anhui Academy of Agricultural Sciences, Hefei 230031, China; 4 Beijing Engineering Research Center on Animal Healthy Environment, Beijing 100083, China; 5 Beijing Honneur Agriculture and Technology Ltd., Beijing 101101, China

**Keywords:** calf, automated milk feeder, slightly acidic electrolyzed water, response surface methodology, bacterial contamination, cleaning efficacy

## Abstract

Automated milk feeders (AMFs) offer significant advantages in promoting natural feeding behavior in calves and reducing manual labor. With widespread use, the impact of AMF hygiene on calf health has attracted increasing research attention, as inadequate cleaning protocols may lead to bacterial accumulation on calf-contact surfaces and subsequent health risks for calves. This study aimed to quantify bacterial contamination on AMF surfaces, evaluate the cleaning efficacy of slightly acidic electrolyzed water (SAEW) compared to warm water and chemical disinfectants (*n* = two total samples), and optimize SAEW cleaning parameters using response surface methodology (RSM). Results revealed that bacterial loads on five high-frequency calf-contact surfaces ranged from 5.48 to 8.21 log_10_ CFU/m^2^. SAEW at 60 mg/L achieved significantly higher cleaning efficacy than warm water and chemical disinfectants under field conditions (*p* < 0.01). Through RSM optimization (highly significant (*p* < 0.001)), the optimal SAEW cleaning parameters were determined as follows: cleaning time of 35 s, cleaning temperature of 78 °C, and available chlorine concentration (ACC) of 108 mg/L. Under the optimized parameters, bacterial and adenosine triphosphate (ATP) removal rates reached approximately 98%. These findings suggest that SAEW is a promising alternative sanitation agent for AMFs, provide preliminary parameters for rapid sanitation under the tested conditions, and hold the potential to support the standardized hygiene control of calf feeding equipment.

## Simple Summary

Automated milk feeders (AMFs) are widely used on dairy farms to help calves feed naturally while reducing manual labor. However, if the feeders are not cleaned properly, harmful bacteria can build up on the surfaces that calves touch, leading to calf illness. This study quantified bacterial loads on key surfaces of feeders, compared slightly acidic electrolyzed water (SAEW) with chemical disinfectants, and optimized sanitation process parameters via response surface methodology. We found bacterial loads on the feeder’s baffle, fence, and teat ranged from 5.48 to 8.21 log_10_ CFU/m^2^, far exceeding the hygienic standard for calf feeding equipment. We identified optimal parameters: 35 s of cleaning time, 78 °C cleaning temperature, and 108 mg/L of available chlorine concentration, which removed 98% of bacteria. With optimal sanitation parameters, SAEW is effective and compatible with AMF applications. Our findings provide a technical reference for SAEW as a promising alternative sanitation agent for AMFs, offer a basis for standardized hygiene management of calf feeding equipment, and hold potential to support more sustainable dairy farming practices.

## 1. Introduction

Calves represent the critical future foundation of the dairy industry, and early-life management directly influences morbidity, mortality, and lifetime productivity [1,2]. Effective feeding management is essential for meeting nutritional requirements while minimizing disease risk. Automated milk feeders (AMFs) are increasingly adopted in group-housing systems, delivering milk via feeding stations that allow self-regulated intake according to individual calf demand [3]. This technology supports higher feeding frequencies, extended milk access periods, and reduced labor dependency [4], thereby improving both calf welfare and farm operational efficiency.

However, the hygiene management of AMFs presents a critical challenge [5,6]. The complex piping systems and fittings, combined with nutrient-rich milk residues and favorable temperatures, create conditions conducive to the proliferation of spoilage bacteria and pathogens [7,8]. As calves frequently lick and suck the external surfaces of AMFs during both rewarded and unrewarded visits [9], the shared teat and milk lines can function as reservoirs and transmission hotspots for infectious agents, significantly increasing the risk of cross-contamination among group-housed animals [10]. Unfortunately, most built-in AMF cleaning programs address only internal circuits, while external contact surfaces and adjacent fixtures often lack routine sanitation. Survey data indicate that approximately half of farms lack regular cleaning procedures for AMF external surfaces, and fewer than 20% employ disinfectants for this purpose [11]. This inadequate attention to AMF hygiene has been identified as a contributing factor to enteric disease incidence in calves [12]. Young calves typically have immature immune systems, so maintaining clean and hygienic automated feeding equipment surfaces is critical to preventing pathogen spread and ensuring calf health [13,14].

Among farms that implement sanitation protocols, chemical agents remain the predominant choice due to their established efficacy and broad-spectrum activity. However, these agents have been criticized for generating harmful by-products, leaving chemical residues, and potentially contributing to antimicrobial resistance [15,16]. Furthermore, detergents containing strong acids and alkalis are highly caustic and may cause serious burns upon contact with skin and mucous membranes [17]. Slightly acidic electrolyzed water (SAEW), produced by electrolysis of dilute NaCl solution, has emerged as a promising alternative. SAEW offers broad-spectrum antimicrobial activity, non-toxicity, environmental friendliness, and the absence of harmful residues [18,19]. The bactericidal mechanism of SAEW relies on the strong oxidizing properties of active chlorine species, primarily hypochlorous acid (HClO), which effectively inactivates microorganisms by disrupting cell membrane integrity and oxidizing intracellular components [20]. The slightly acidic pH (5.0–6.5) of SAEW maintains HClO as the predominant active species.

As an efficient disinfectant, SAEW has been widely used in livestock and poultry farming, with excellent sanitation efficacy demonstrated in drinking water line treatment [21], plastic transport cage sanitization [22], and pathogen inactivation in various livestock facilities [23,24,25,26]. It has also exhibited superior and long-lasting bacterial control over conventional chemical disinfectants [21]. However, the optimal SAEW cleaning and sanitation parameters vary significantly by target surface material, contamination type, and operational constraints. Liu et al. [27] reported optimal parameters of 9.9 min at 37.8 °C with 60 mg/L available chlorine concentration (ACC) for stainless steel milking system surfaces, while Dev et al. [28] recommended a 10 min treatment at 39.8 °C for acidic electrolyzed water. Such extended cleaning periods are not feasible for AMFs, given the frequent feeding behavior of calves and the need to minimize equipment downtime.

To investigate the sanitation efficacy of SAEW and optimize parameter settings, this study was based on the core hypothesis that SAEW achieves superior sanitation efficacy on AMF surfaces compared with conventional agents, and that SAEW cleaning parameters optimized via response surface methodology (RSM) can achieve efficient, rapid decontamination for AMFs. This study aimed to: (1) quantify bacterial contamination on AMF surfaces under field conditions; (2) compare the cleaning efficacy of SAEW (at different ACCs) with warm water and chemical disinfectants under field conditions; (3) optimize SAEW sanitation parameters (time, temperature, ACC) via RSM in the laboratory optimization experiment to establish the optimal parameters for AMFs. The optimized parameters were developed to meet the rapid cleaning demands of frequent calf feeding schedules, while supporting fast, effective cleaning for other livestock feeding equipment. This study provides basic data for SAEW application in AMF hygiene management to improve calf health and welfare in group-housed calf systems.

## 2. Materials and Methods

### 2.1. Preparation of Cleaning Agents

The SAEW were prepared using the Harmony Oxidation Potential System (model: Anywhere-320W, Beijing Rui’ande Environment Technology Co., Ltd., Beijing, China). SAEW with ACCs of 40 mg/L, 50 mg/L, 60 mg/L, 90 mg/L, 105 mg/L and 120 mg/L were prepared by adding NaCl to tap water, adjusting the outlet of the acidic electrolyzed water, and regulating the current in the electrolysis equipment. The ACC was determined by a digital chlorine test kit (model: RC-2Z, Kasahara Chemical Instruments Co., Saitama, Japan). The pH value of the SAEW was monitored using a benchtop pH meter (model: PH-8414, Hangzhou Ying’ao Technology Co., Hangzhou, China) and maintained between 5.5 and 6.5 for all treatment groups. The chemical disinfectant was an acidic cleaner containing H_2_SO_4_ and HCl (pH 2.1), prepared at 0.5% (*v*/*v*) by diluting the concentrated detergent (model: Cidmax, DeLaval Co., Ltd., Tianjin, China) with tap water, following manufacturer instructions. Both the SAEW and chemical disinfectant were freshly prepared within 30 min before use.

### 2.2. Field Experiment Design

#### 2.2.1. Experimental Site and AMF Equipment

The field experiment was conducted on a commercial dairy farm in Hebei, China, housing approximately 1000 dairy cows including 96 calves managed under a group-housing system. AMFs (Lely Co., Ltd., Qingdao, China) were used for calf rearing. One calf pen, housing 15–20 pre-weaned calves, was selected for the study. The pen was 12 m × 12 m, divided into a feeding area (12 m × 2 m) and a bedding area (12 m × 10 m). The AMF was installed in the feeding area, allowing calves to feed freely.

The AMF performs clean-in-place (CIP) procedures twice a day, sanitizing the interior of the feeding unit and internal hoses with chemical disinfectant and warm water; each cycle lasts 3 min. The teats are disassembled weekly for soaking and manual cleaning. The external surfaces of the AMF and surrounding fences do not undergo additional cleaning.

#### 2.2.2. Sampling Sites

**Figure 1:**
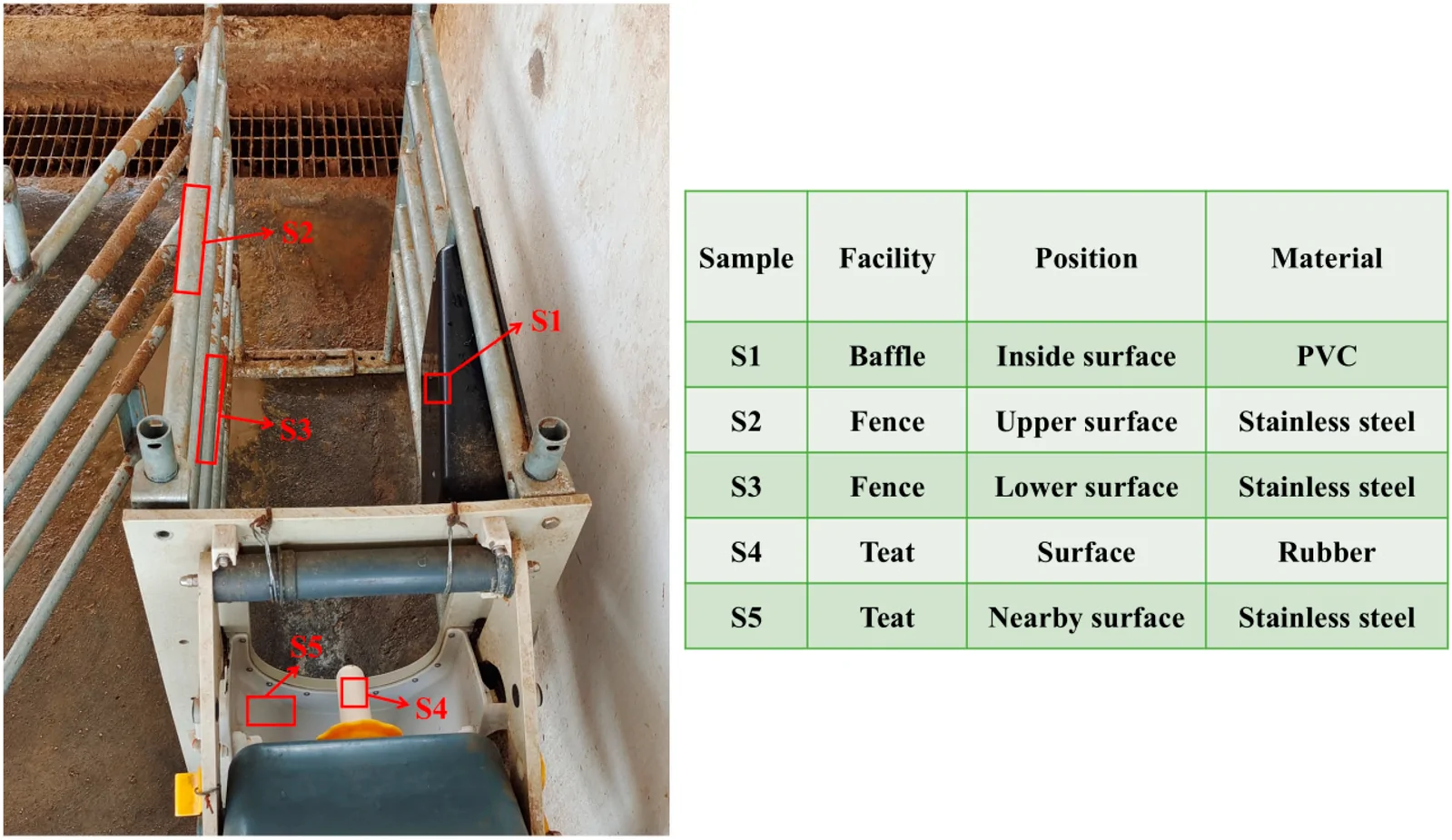
Sampling sites (S1–S5) frequently licked by calves on the AMF and surrounding fences.

Preliminary on-site observations revealed that calves frequently exhibited licking behavior on the surfaces of the AMF baffle, surrounding fences, and teat units before and after feeding. Based on these observations, five sampling sites (S1–S5) were selected from surfaces with high licking frequency (Figure 1). The sites included: S1, inner surface of the AMF baffle (PVC); S2, upper surface of the surrounding fence (stainless steel); S3, lower surface of the surrounding fence (stainless steel); S4, surface of the teat (rubber); S5, nearby surface of the teat (stainless steel).

#### 2.2.3. Cleaning Treatments

A randomized complete block design was used in the field experiment, with treatment day as the blocking factor. Six treatments were evaluated: no cleaning (blank control), warm water cleaning, chemical disinfectant cleaning, and SAEW cleaning at ACCs of 40, 50, and 60 mg/L. The temperature of all cleaning agents was maintained at 45 °C. One treatment was applied to all five sampling sites per day, and each treatment was implemented on two independent days in a completely random order across two experimental cycles (12 days total).

**Figure 2:**
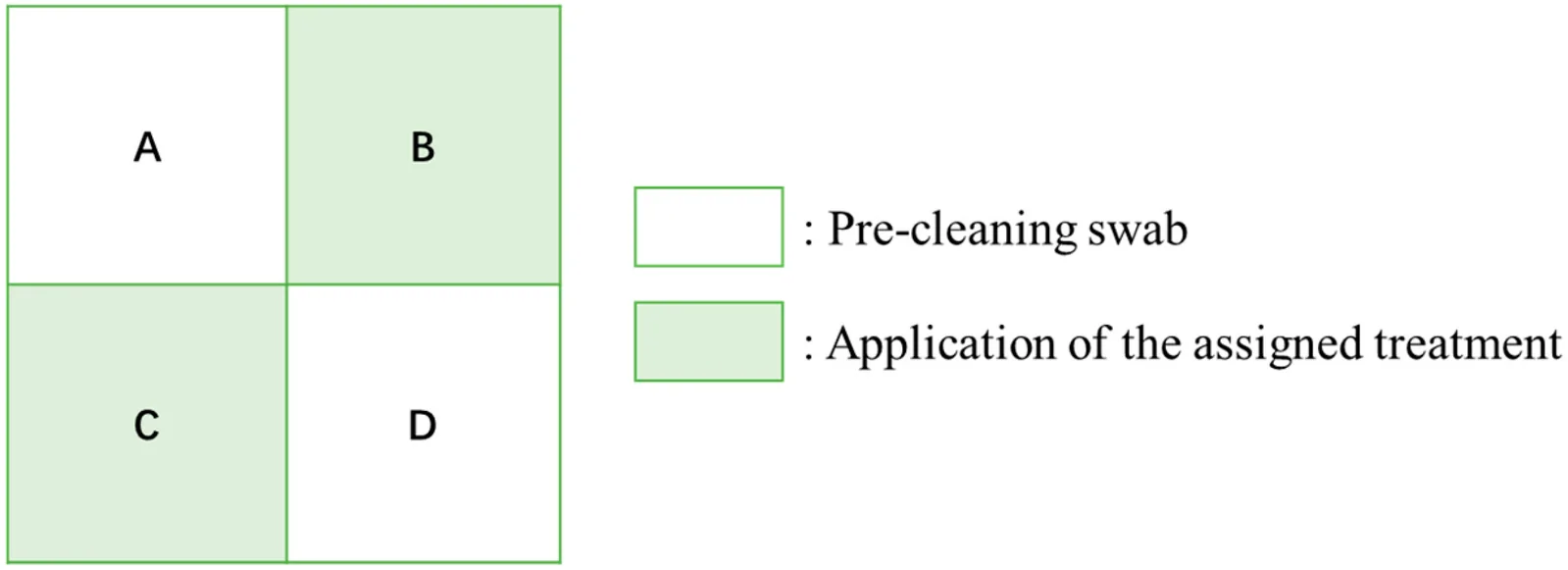
Schematic diagram of the sampling template and paired sampling strategy. Note: Window A: Pre-cleaning swab sampling location for Pair 1; Window B: Post-cleaning swab sampling location (adjacent to D) for Pair 2; Window C: Post-cleaning swab sampling location (adjacent to A) for Pair 1; Window D: Pre-cleaning swab sampling location (adjacent to B) for Pair 2.

A paired sampling strategy was designed to eliminate the influence of spatial heterogeneity of surface contamination on cleaning efficacy evaluation. At each sampling site, a sterile portable template defining a 3 × 3 cm (9 cm^2^) micro-area was used for standardized sampling. The template contained four equal, non-overlapping square windows (Figure 2A–D). Two independent pre- and post-cleaning paired samples were collected for each treatment. Pair 1: pre-cleaning swab sampling from window A; following application of the assigned cleaning treatment, post-cleaning swab sampling from the adjacent window C. Pair 2: pre-cleaning swab sampling from window D; following application of the same treatment, post-cleaning swab sampling from the adjacent window B. All windows were positioned within the same micro-area to ensure baseline contamination homogeneity, while the non-overlapping design completely avoided cross-contamination between pre- and post-cleaning sampling. For S4, a flexible split-ring silicone template with two 1.5 × 1.5 cm windows was used. During the entire sampling and cleaning process, a dedicated handler kept calves away from the test area for approximately 10–15 min, and normal calf access to the pen was restored immediately after all operations were completed. All templates and disposable nitrile gloves were changed between sites to prevent cross-contamination.

The standardized cleaning procedure was performed as follows: cleaning solutions were applied using calibrated spray bottles at a fixed dose of approximately 22.5 µL (10 µL/cm^2^). The cleaning agent was left to act on the surface for a standardized contact time of 60 s, then the target surface was wiped with sterile single-use microfiber cloths using 10 horizontal and 10 vertical strokes under consistent moderate pressure.

#### 2.2.4. Sample Collection

Surface samples were collected using sterile medical cotton swabs pre-moistened with sterile buffered peptone water (BPW). For each sampling window, the swab was rubbed with 10 horizontal and 10 vertical passes while continuously rotating the swab tip to ensure full coverage of the entire target area. After sampling, the swab head was aseptically detached into a sterile test tube containing 10 mL sterile BPW. All samples were stored at 0–4 °C immediately after collection and transported to the laboratory for subsequent microbial analysis, as described in Section 2.4.

### 2.3. Laboratory Optimization Experiment

#### 2.3.1. Experimental Setup and Bacterial Inoculation

The bacterial strains used in the laboratory experiments were three dominant contaminant isolates (*Escherichia coli*, *Staphylococcus aureus* and *Salmonella* spp.) from raw milk and equipment surface samples of the target dairy farm, which were isolated and identified by a third-party microbial testing institution with CMA accreditation. All strains were stored in glycerol stocks at −80 °C until use.

A mixed inoculum composed of the above three strains was used to simulate the actual on-farm contamination scenario. Each strain was individually cultured in tryptic soy broth at 37 °C for 24 h, and the bacterial concentration was adjusted to approximately 1 × 10^9^ CFU/mL via plate counting. Equal volumes of each single-strain suspension were thoroughly mixed to prepare the standardized mixed inoculum. Raw milk for calf feeding was autoclaved at 121 °C for 15 min to eliminate background microflora before use. An aliquot of 1 mL of the mixed inoculum was centrifuged (4 min, refrigerated benchtop centrifuge), the resulting pellet was washed twice with 0.1% BPW, and resuspended in 10 mL sterilized raw milk to yield a final bacterial concentration of 1 × 10^8^–1 × 10^9^ CFU/mL.

Test specimens (3 cm × 3 cm) were prepared from stainless steel and PVC, which are the two most commonly used materials for AMF surfaces. Eighteen specimens per material were randomly allocated to three groups (*n* = six per group): blank controls (no inoculation, no cleaning), initial contamination control (inoculation, no cleaning), and SAEW treatment group (inoculation and cleaning treatment). All specimens were autoclaved at 121 °C for 20 min and air-dried under sterile conditions before use. For inoculation, 100 µL of the contaminated raw milk was pipetted onto each specimen within a laminar flow cabinet and spread evenly using a sterile glass spreader. Inoculated specimens (six initial control and six treatment specimens) were air-dried under sterile conditions for approximately 2 h to allow bacterial attachment to the surface.

SAEW cleaning was performed following the same manual spraying and wiping protocol used in the field experiment. Contaminated specimens were placed horizontally, sprayed with 90 µL (10 µL/cm^2^) of cleaning solution, and wiped using a standardized motion. For each material, six treatment specimens were equally divided into two parallel test groups (*n* = three per group): three for bacterial reduction rate analysis and three for ATP measurement, to ensure result reliability and repeatability. Similarly, the six initial contamination control specimens were split into two matched triplicate sets for corresponding baseline bacterial count and ATP detection, respectively. After cleaning, the entire surface of the specimen was swabbed with sterile cotton swabs moistened in 0.1% BPW, following the same sampling procedure described in Section 2.2.4. Swabs were transferred into tubes containing 10 mL 0.1% BPW for subsequent microbial analysis, as described in Section 2.4.

#### 2.3.2. Single-Factor Experiments

A series of preliminary single-factor trials were conducted to determine the feasible range of three SAEW cleaning parameters: cleaning time, cleaning temperature, and ACC (Table 1). In each single-factor trial, two parameters were held at intermediate values while the third was varied from low to high; responses were measured as bacterial removal rate and ATP removal rate on stainless steel and PVC surfaces. Based on these results, the final ranges were identified as cleaning time 30–40 s, cleaning temperature 65–80 °C, and ACC 90–120 mg/L.

#### 2.3.3. Box–Behnken Response Surface Design

To optimize SAEW cleaning parameters while minimizing the number of trials, a three-factor Box–Behnken response surface design was employed. A standard Box–Behnken design for three variables requires 15 experimental trials, consisting of 12 factorial runs and three center point runs (Table 2). Three center point runs with all factors fixed at the intermediate level were evenly interspersed at the start, middle, and end of the entire experimental sequence. Although response surface experiments are ideally performed in random order, trials in this study were grouped by temperature to reduce adjustments to the water bath, which would otherwise cause temperature overshoot and unstable fluctuation. Three center point runs could verify the stability of the experimental system and test for potential run-order effects.

### 2.4. Evaluation Methods

Contamination levels and cleaning efficacy were assessed using two complementary methods: aerobic plate count (APC) and ATP bioluminescence assay.

#### 2.4.1. Aerobic Plate Count

APC provides direct quantification of viable bacteria and serves as the primary indicator of bactericidal efficacy. Swab suspensions were serially diluted tenfold in sterile 0.85% saline using calibrated micropipettes. Aliquots of 0.1 mL from appropriate dilutions were spread-plated in duplicate onto plate count agar (PCA). Plates were incubated aerobically at 37 °C for 48 h. Colonies were counted manually, and only plates with 30–300 colonies were used for calculation. Bacterial concentration was calculated according to Equation (1):

(1) $$P=\frac{50N10^{x}}{s},$$

where *P* is the bacterial count (CFU/cm^2^), *N* is the number of colonies on the plate (CFU), *x* is the dilution factor, and *S* is the specimen surface area (cm^2^).

#### 2.4.2. ATP Bioluminescence Assay

ATP bioluminescence detects both microbial and non-microbial organic residues, making it suitable for evaluating overall surface cleanliness [29,30]. ATP levels were measured using a handheld luminometer (Luminator-T CF-420, Shanghai Canfu Jidian Co., Shanghai, China) following the manufacturer’s protocol. Results were expressed as relative light units (RLUs).

#### 2.4.3. Calculation of Removal Rates

Bacterial removal rate and ATP removal rate were set as the response variables for the optimization of SAEW cleaning parameters, and both were calculated as percentage removal using the following formulas:

(2) $$Bacteria\;removal\;rate\left(\%\right)=(\frac{B_{0}-B_{1}}{B_{0}})\times 100,$$

(3) $$ATP\;removal\;rate(\%)=(\frac{A_{0}-A_{1}}{A_{0}})\times 100,$$

where $B_{0}$ and $A_{0}$ are the initial bacterial count (CFU/cm^2^) and ATP level (RLU) of the surface before cleaning, and $B_{1}$ and $A_{1}$ are the bacterial count (CFU/cm^2^) and ATP level (RLU) on the surface after cleaning, respectively.

### 2.5. Response Surface Modeling and Optimization

Separate second-order polynomial response surface models were developed for bacterial and ATP removal rates to characterize the relationship between the independent variables (cleaning time, cleaning temperature, ACC) and the two response variables. The general form of the quadratic polynomial model is as follows:

(4) $$R=\sum_{i=1}^{3}\left(a_{i}x_{i}^{2}+c_{i}x_{i}\right)+\sum_{i=1}^{3}\sum_{j=1}^{3}b_{ij}x_{i}x_{j}+C\cdot (i\neq j),$$

where *R* denotes the response value (bacteria removal rate or ATP removal rate), $x_{i}$ are the coded factor levels of the independent variables, $a_{i}$ , $b_{ij}$ , and $c_{i}$ are the quadratic, interaction, and linear coefficients, respectively, and C is the intercept.

Multi-objective optimization was performed using the equal-weight linear aggregation method, with the core objective of maximizing the comprehensive cleaning efficiency (CCE) of SAEW. The bacterial removal rate and ATP removal rate were deemed equally important for cleaning performance evaluation; both indicators were calculated as percentage removal, so no additional normalization was required to eliminate dimensional and magnitude differences. The composite objective function is defined as follows:

(5) $$maxCCE=\frac{R_{ba}+R_{ATP}}{2}s.t.x_{i}\in \Omega_{i},$$

where *CCE* is the comprehensive cleaning efficiency, serving as the unified optimization objective; $R_{ba}$ is the predicted bacterial removal rate (%) from the established response surface model; $R_{ATP}$ is the predicted ATP removal rate (%) from the established response surface model; $x_{i}$ is the i-th independent variable; $\Omega_{i}$ is the set experimental range of the i-th independent variable.

On the basis of maximizing the CCE value, the optimal solution with the minimum normalized sum of independent variables was selected as the final optimized cleaning protocol, to achieve the optimal cleaning efficacy with the lowest input of cleaning parameters. The optimization procedure was implemented using the Response Optimizer module in Minitab software (https://www.minitab.com/en-us/). The optimization steps were as follows: (1) set the constraint ranges of all independent variables to be completely consistent with those in the response surface experimental design; (2) set the optimization goal of both the bacterial removal rate and ATP removal rate to “maximize”, with equal weight assigned to both responses; (3) solve the feasible solution set with the highest predicted CCE value via the built-in desirability function algorithm of the software; (4) from the above feasible solution set, select the solution with the minimum normalized sum of independent variables as the final optimized cleaning protocol. Triplicate verification experiments were conducted to validate the accuracy and reliability of the optimized protocol.

### 2.6. Statistical Analysis

All statistical analyses were performed using SPSS 21.0 (SPSS Inc., Chicago, IL, USA) and Minitab 17 (Minitab, Inc., State College, PA, USA). SPSS Statistics was used for the statistical analysis of data from field trials, single-factor trials and validation trials, while Minitab was exclusively employed for the experimental design, model fitting, and parameter optimization of the RSM experiment. Results are expressed as mean ± standard error of the mean. Statistical significance was set at *p* < 0.05.

#### 2.6.1. Statistical Analysis for the Field Experiment

Field experiments were conducted with two independent biological replicates (treatment days) per treatment, limited by practical on-farm constraints, and each sampling site was paired with two technical replicates (paired sampling windows). Prior to model fitting, the two technical replicates from the same sampling site × treatment day combination were averaged to yield a single representative value for each sampling site per day.

Field experimental data were analyzed using a linear mixed model (LMM) to account for the nested design structure and non-independence of observations. Bacterial counts were log_10_ transformed. Normality of conditional residuals was verified by the Shapiro–Wilk test, and homogeneity of residual variances was assessed via visual inspection of residual plots. For the fixed effects in the LMM, a type III F-test was used to assess overall significance. Fisher’s LSD test was used for post hoc pairwise multiple comparisons between treatment groups. Fixed effects included treatment, sampling site, and their interaction, as well as the pre-treatment baseline value as a covariate. Day and sampling site nested within day were defined as random effects. Day was included as a random blocking factor to account for uncontrolled environmental variation between independent experimental days.

#### 2.6.2. Statistical Analysis for the Laboratory Optimization Experiment

All laboratory validation experiments were performed in triplicate. Arcsine square-root transformation was performed for bacterial and ATP removal rates. Normality of distribution was verified via the Shapiro–Wilk test, and homogeneity of variances was confirmed via Levene’s test prior to formal statistical analysis. One-way ANOVA was used to evaluate the effect of each parameter level on the bacterial and ATP removal rates for the single-factor laboratory experiment. For the BBD experiment, multiple regression analysis was performed to fit the second-order polynomial model (Equation (4)) to the experimental data. The significance of the linear, quadratic, and interaction terms of the model was assessed using a F-test. The goodness of fit of the model was evaluated using the coefficient of determination (R^2^), adjusted R^2^, and lack-of-fit test.

Paired *t*-tests were used to verify model prediction accuracy by comparing observed and model-predicted response values, and to confirm consistent sanitization efficacy between stainless steel and PVC surfaces. The optimal sanitation parameters were obtained via Minitab 17 numerical optimization (Section 2.5).

Pearson correlation coefficients were calculated to evaluate the linear relationship between bacterial removal rate and ATP removal rate. Significance testing for the correlation coefficient was performed using a *t*-test, and 95% confidence intervals were also calculated.

## 3. Results and Discussion

### 3.1. Microbial Contamination and Cleaning Efficacy in the Field Experiment

**Figure 3:**
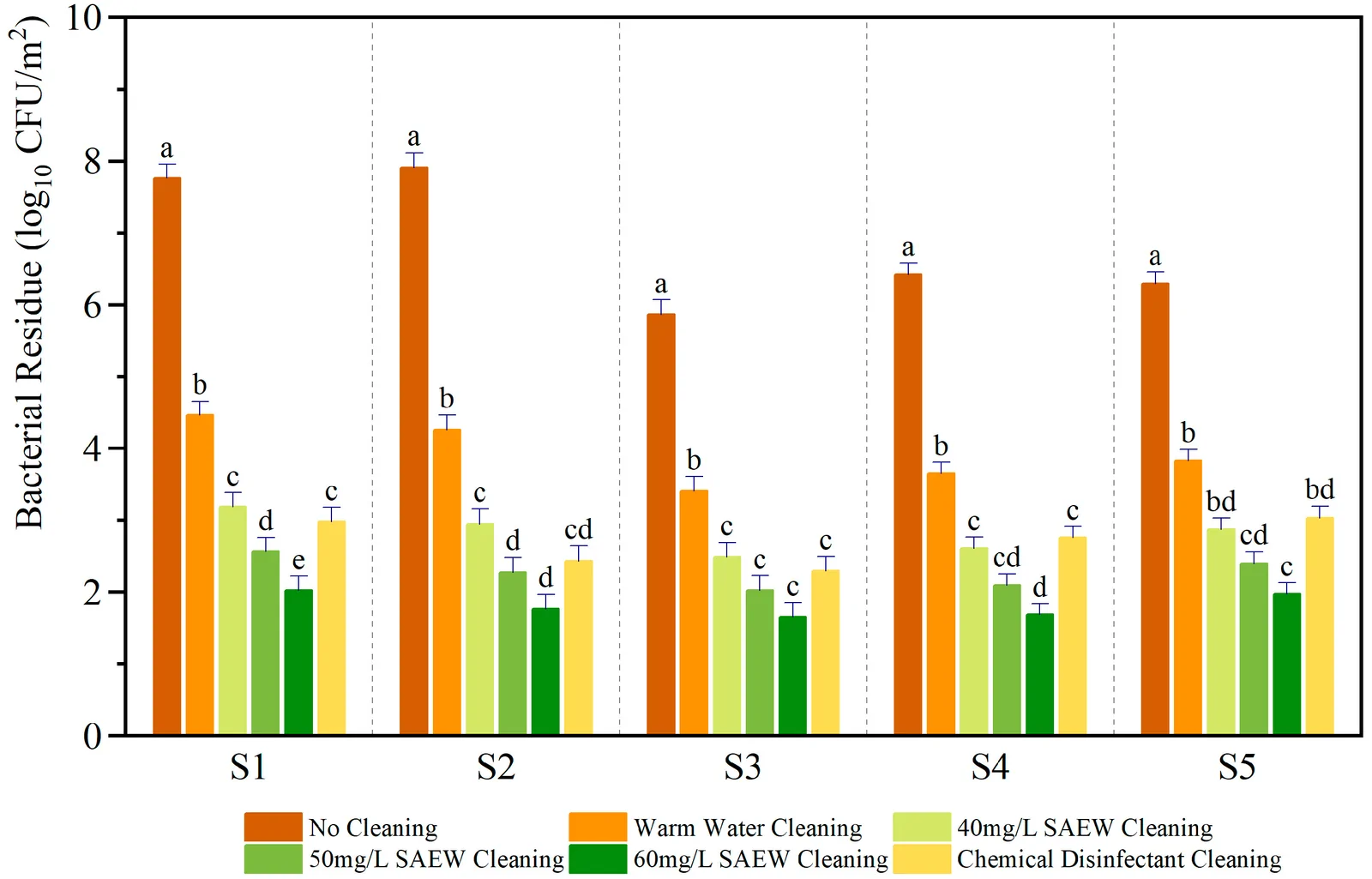
The bacterial residues on frequent calf-lick positions with five different cleaning agents. *n* = 2 biological replicates for each treatment group at each sampling site. Note: Different lowercase letters above bars indicate significant differences between treatments within the same sampling site (LSD test, *p* < 0.05).

The bacterial residues before and after cleaning with different treatments are presented in Figure 3. The LMM revealed a highly significant main effect of cleaning treatment (*F* = 253.268, *p* < 0.001), sampling site (*F* = 18.498, *p* < 0.001), and treatment × site interaction effect (*F* = 16.133, *p* < 0.001). The covariate pre-cleaning showed a marginally significant effect (*p* = 0.078), indicating a trend of baseline contamination level influencing post-cleaning bacterial residues.

#### 3.1.1. Baseline Contamination Levels on AMF Surfaces

Prior to cleaning, total viable count (TVC) ranged from 5.58 to 8.21 log_10_ CFU/m^2^ across the five sampling sites. Although no specific regulatory threshold exists for acceptable bacterial loads on AMF surfaces, Böhm [31] proposed a general hygiene target of ≤3 log_10_ CFU/cm^2^ (equivalent to ≤4.48 log_10_ CFU/m^2^) for surfaces in contact with animals following prophylactic sanitation. The high baseline bacterial counts observed in this study (5.58 to 8.21 log_10_ CFU/m^2^) clearly indicate substantial microbial contamination that far exceeds this acceptable hygiene standard. These findings underscore the critical importance of implementing regular and effective cleaning protocols for AMFs to minimize pathogen exposure and protect calf health.

#### 3.1.2. Comparative Efficacy of Different Cleaning Agents

The cleaning efficacy of the six treatments varied significantly, with a highly significant main effect of treatment (*p* < 0.001). Based on bacterial reduction efficiency and post hoc LSD test results, the effectiveness of the treatments generally followed the order: 60 mg/L SAEW > 50 mg/L SAEW > chemical disinfectant/40 mg/L SAEW > warm water cleaning. The relative efficacy of the chemical disinfectant versus 40 mg/L SAEW differed across sampling locations, with no consistent advantage for either treatment.

Cleaning with warm water reduced TVC by approximately 1–3 log units; however, residual counts remained at 3.12–4.72 log_10_ CFU/m^2^, with most values still exceeding the acceptable hygiene threshold. This indicates that warm water alone provides insufficient sanitizing efficacy for AMF surfaces and cannot meet the hygiene requirements of calf feeding facilities.

SAEW treatments demonstrated ACC-dependent bactericidal efficacy. At 40 mg/L ACC, bacterial reductions of 3.43–4.96 log units were achieved, with residual counts of 2.82 ± 0.52 log_10_ CFU/m^2^. The 60 mg/L SAEW treatment achieved the highest bactericidal efficacy across all sites, with residual bacterial counts consistently ≤2.30 log_10_ CFU/m^2^, corresponding to 4.21–6.15 log reductions compared to baseline levels. Cleaning efficacy was significantly higher at an ACC of 60 mg/L than at 40 mg/L (*p* < 0.001), while no significant differences were observed between 40 and 50 mg/L (*p* > 0.05), as well as between 50 and 60 mg/L (*p* > 0.05). This ACC-dependent relationship is consistent with the established oxidative mechanism of HClO, which exerts antimicrobial effects through disruption of bacterial cell membrane integrity, oxidation of functional proteins, and inactivation of intracellular enzymes [32,33]. Higher ACC provides greater oxidative capacity, thereby accelerating these bactericidal processes and achieving more rapid microbial inactivation. This ACC–response relationship has been consistently reported in previous studies. Rahman et al. [34] demonstrated that increasing ACC from 20 to 50 mg/L significantly enhanced the reduction in Escherichia coli and Salmonella on fresh produce surfaces. Similarly, Hao et al. [35] reported that SAEW with an ACC of 60 mg/L achieved >5 log_10_ CFU reduction in pathogenic bacteria on poultry processing equipment, compared to 3–4 log reduction at 30 mg/L.

The commercial chemical disinfectant achieved bacterial reductions of 3.27–5.47 log units, with residual counts of 2.70 ± 0.45 log_10_ CFU/m^2^. The efficacy of the chemical disinfectant was statistically comparable to that of SAEW at 40 or 50 mg/L ACC (*p* > 0.05), but significantly lower than that of SAEW at 60 mg/L ACC (*p* = 0.004). Although after using chemical disinfectant, the residual bacterial counts at most sampling sites met the acceptable hygiene standard [31], chemical disinfectants may present practical limitations, including corrosivity to skin and equipment [36,37,38] and potential risks from chemical residues on feeder surfaces [39,40]. These safety concerns limit the suitability of chemical disinfectants for routine AMF sanitation.

#### 3.1.3. Effect of Surface Characteristics and Materials on AMFs

Differences among sampling sites revealed site-specific variation in contamination level and cleanability, which can be explained by calf-contact behavior, environmental exposure, and surface characteristics. The inner surface of the baffle (S1) and upper surface of the fence (S2) showed the highest baseline contamination and post-cleaning residues, followed by the teat contact area (S4 and S5). Although the teat was in direct contact with calves’ mouths during feeding and served as the primary site for cross-contamination through repeated licking, the bacterial counts on the teat surface were not the highest among all sampled sites, in contrast to findings reported by Heinemann et al. [10]. Several factors may explain this discrepancy. First, the farm implemented a weekly protocol of manual disassembly and soaking cleaning for teats, and our field sampling was conducted mid-cycle between cleaning events, which likely reduced bacterial accumulation. Second, the frequent mechanical rinsing from calf suckling, together with continuous saliva flow, may have exerted a natural cleaning effect [36]. Furthermore, saliva contains antimicrobial components such as lysozyme and lactoferrin, which could have inhibited bacterial colonization on the teat surface [37,38].

The high bacterial loads at S1 and S2 can be attributed to multiple interacting factors. During the observation period, calves frequently exhibited licking and touching behaviors at these sites before and after AMF use, leading to bacterial contamination from oral cavity and nasal secretions [39,40]. The moist environment created by saliva deposition provided favorable conditions for bacterial proliferation and biofilm formation [41,42]. Additionally, these sites were exposed to the external environment and prone to accumulating dust, feed residues, and organic matter, which served as substrates for bacterial attachment and growth [43]. The combination of frequent animal contact, moisture retention, and organic matter accumulation created a microenvironment highly conducive to bacterial colonization and persistence. In contrast, site S3 (the lower surface of the fence) exhibited the lowest bacterial counts, as its position below the typical height accessible to calves reduced direct contact frequency. These findings highlight the importance of prioritizing sites with frequent animal contact and moisture accumulation during cleaning procedures, like the inner walls and the upper barrier of the AMF.

Surface materials also influence biofilm formation [42] and cleaning efficacy of SAEW [44]. Softer materials prone to surface scratches facilitate bacterial attachment [45,46], and rough surfaces are more difficult to clean than smooth surfaces [47,48]. Although the direct relationship between material type and bacterial load or cleaning efficacy was not investigated in this study, we still recommend that smooth, hard-surfaced materials be preferentially used for AMFs. Soft materials such as PVC and rubber should be regularly cleaned and replaced to minimize bacterial colonization. Since rubber is used only for teats in small scale and exhibits similar cleaning parameters to PVC [27], it was excluded from parameter optimization in the laboratory experiment.

### 3.2. Response Surface Optimization of SAEW Parameters

#### 3.2.1. Experimental Results and Data Overview

Based on the Box–Behnken design presented in Table 2, the bacterial removal rate and ATP removal rate under different treatments are summarized in Table 3.

One-way ANOVA results of the three center point runs evenly interspersed throughout the entire experimental sequence showed that there was no significant difference in the response values of the center points conducted at different stages of the experiment (bacterial removal rate: *p* = 0.300 > 0.05; ATP removal rate: *p* = 0.489 > 0.05), indicating that the temperature-based grouping of experimental runs in this study did not introduce significant time-related or run-order-related effects, and the results of the response surface model were reliable.

SAEW demonstrated consistently high cleaning efficiency across all parameter combinations, with bacterial removal rates of 82.8–100.0% (mean: 94.67 ± 5.1%) and ATP removal rates of 82.1–100.0% (mean: 94.2 ± 5.9%). The highest cleaning efficiency was observed in groups 4 and 15, which achieved complete removal for both indicators under higher ACC or temperature combined with longer cleaning time. The lowest removal rates were recorded in group 8 (bacterial: 82.8%; ATP: 82.1%), corresponding to lower ACC and temperature. These preliminary observations suggest that ACC and temperature may be important factors influencing SAEW efficacy, which will be further examined through response surface analysis. For comparison, the chemical disinfectant achieved bacterial and ATP removal rates of only 76.0% and 72.0%, lower than all SAEW treatment groups (*p* < 0.05). As expected, no measurable removal was observed in the uncleaned control group.

Paired samples *t*-tests were performed to compare bacterial and ATP removal rates between stainless steel and PVC surfaces across all treatment groups. As shown in Table S1, no significant differences were detected for bacterial removal rate (*p* > 0.05) or ATP removal rate (*p* > 0.05). Accordingly, the mean removal rates from the two materials were used as unified response values for RSM model fitting. This approach ensured the interpretability of the optimization model and enabled the development of a single, universal optimized protocol applicable to both of the two most common materials used in AMFs. Given that these surfaces are in frequent direct oral contact with calves, the consistent high-efficacy decontamination achieved across both materials across all RSM experimental parameter ranges can minimize microbial exposure risk for immunologically immature young calves, while also enhancing the on-farm practicality of the optimized sanitation protocol.

ATP content reflects both microbial load and organic residues, which is particularly relevant for AMF sanitation since milk residues can act as a nutrient source for microbial regrowth. A strong positive correlation was observed between bacterial removal rate and ATP removal rate (*r* = 0.98, *p* < 0.001), indicating that ATP bioluminescence can serve as a reliable and rapid indicator for assessing surface hygiene [30,49].

#### 3.2.2. Model Fitting and Adequacy Evaluation

To elucidate the relationships between cleaning parameters and cleaning efficacy, bacterial removal rate and ATP removal rate were fitted to quadratic polynomial models using Equation (4). The ANOVA results are presented in Table 4. Both models were highly significant (*p* < 0.001).

The R^2^ indicates the proportion of variance explained by the model. An adjusted R^2^ higher than 0.90 indicated that no significant terms have been missed by the model [50]. In this study, *R*^2^ and adjusted *R*^2^ values were 99.14% and 97.59% for bacterial removal rate, and 99.35% and 98.19% for ATP removal rate, respectively, indicating excellent model performance. The lack-of-fit test was non-significant for both models (*p* = 0.094 and 0.101, respectively), further confirming adequate model fit [50].

Analysis of individual model terms revealed that all linear coefficients (*x*_1_, *x*_2_, *x*_3_), the quadratic terms *x*_2_^2^ and *x*_3_^2^, and the interaction term *x*_2_*x*_3_ were significant (*p* < 0.05), whereas *x*_1_^2^, *x*_1_*x*_2_, and *x*_1_*x*_3_ were non-significant (*p* > 0.05) (Table 4). Based on *F*-values, the relative importance of factors followed the order: cleaning temperature > ACC > cleaning time for both response variables.

Based on the significance analysis, non-significant terms were removed to obtain simplified models:

*R*_*ba*_ = −552.5 + 3.84*x*_1_ + 10.49*x*_2_ + 3.041*x*_3_ − 0.05818*x*_2_^2^ − 0.00769*x*_3_^2^ − 0.01242*x*_2_*x*_3_, (6)

*R*_*ATP*_ = −502.5 + 1.99*x*_1_ + 10.59*x*_2_ + 2.580*x*_3_ − 0.05908*x*_2_^2^ − 0.00635*x*_3_^2^ − 0.00871*x*_2_*x*_3_, (7)

where *x*_1_ is the cleaning time in s, *x*_2_ is the cleaning temperature in °C, and *x*_3_ is the ACC in mg/L.

Previous studies have identified ACC as the primary determinant of SAEW sanitation efficacy relative to cleaning time [22,51,52,53]; however, these studies did not include temperature as an experimental variable. The present study, by incorporating temperature into the optimization framework, demonstrates that thermal effects can surpass the influence of ACC under the conditions tested. At elevated temperatures, the oxidative potential of HClO, the primary active species in SAEW, may be enhanced due to increased molecular kinetic energy and improved penetration through microbial cell membranes [18,34]. This may accelerate oxidative damage to cellular components, including membrane lipids, proteins, and nucleic acids. Additionally, thermal treatment itself may contribute [54]. Furthermore, elevated cleaning temperatures may also reduce the need for the application of physical forces such as water turbulence and scrubbing [42].

#### 3.2.3. Response Surface Plots and Factor Interactions

**Figure 4:**
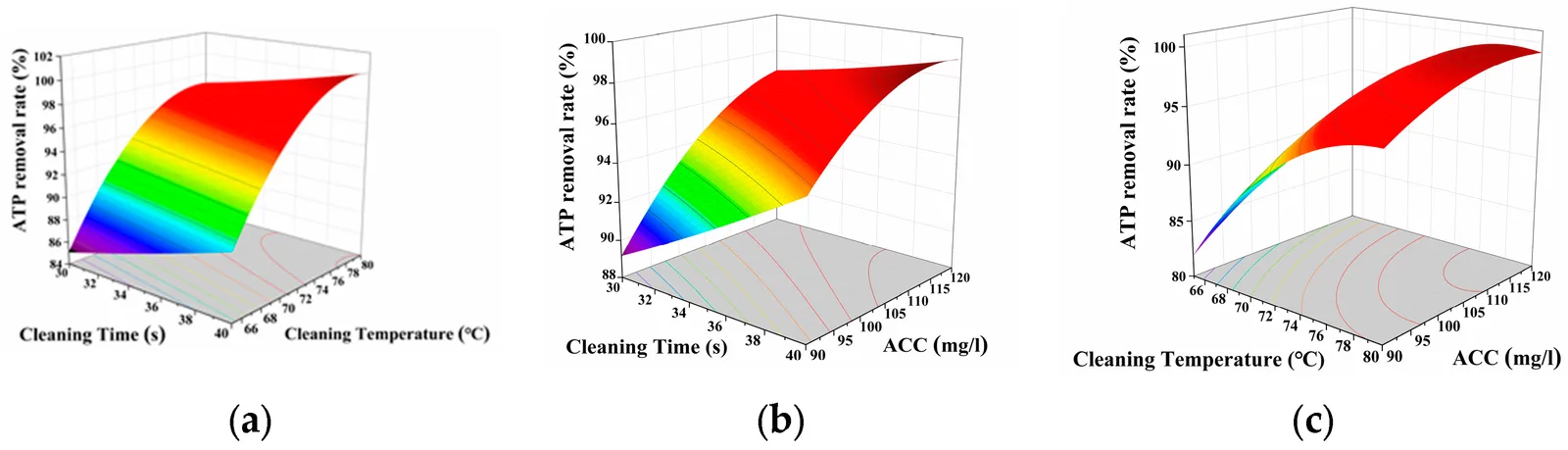
Response surface plots showing the effect of (**a**) cleaning time and cleaning temperature at an ACC of 105 mg/L; (**b**) cleaning time and ACC at a cleaning temperature of 72.5 °C; (**c**) cleaning temperature and ACC at a cleaning time of 35 s on the ATP removal rate.

Based on the strong correlation between bacterial and ATP removal rates (Section 3.2.1) and the superior sensitivity of ATP bioluminescence over traditional plate counting [29], response surface analysis was conducted for ATP removal rate (Figure 4).

Figure 4a shows the combined effects of cleaning time and cleaning temperature at a fixed ACC of 105 mg/L. Cleaning time exhibited a significant positive linear effect (*p* < 0.05), while cleaning temperature showed both a positive linear effect (*p* < 0.001) and a negative quadratic effect (*p* < 0.05) on ATP removal rate. The steeper increase in ATP removal rate with temperature compared to time suggests that elevated temperatures can compensate for shorter cleaning durations. The interaction between cleaning time and cleaning temperature was non-significant (*p* > 0.05).

Figure 4b illustrates the effects of cleaning time and ACC at a fixed cleaning temperature of 72.5 °C. ACC demonstrated a significant positive linear effect (*p* < 0.001) and a negative quadratic effect (*p* < 0.05). At lower ACC levels, ATP removal rate increased gradually with cleaning time; however, at higher ACC levels, the removal rate increased more rapidly, indicating that ACC plays a dominant role in SAEW sanitizing efficacy compared to cleaning time. This observation is consistent with the *F*-value ranking presented in Section 3.2.2.

Figure 4c shows the interaction between cleaning temperature and ACC at a fixed cleaning time of 35 s. Cleaning temperature exerted a more pronounced effect than ACC, and the interaction term (*x*_2_ × *x*_3_) was significant (*p* < 0.05), indicating a synergistic effect between elevated temperature and ACC, which means the bactericidal contribution of higher ACC is enhanced at elevated temperatures. This may be due to higher temperatures increasing the molecular kinetic energy of active chlorine species, thereby enhancing their interaction with surface contaminants and microbial cells [20]. Higher temperatures may increase the decomposition rate, resulting in the observed quadratic relationship (negative *x*_2_^2^ and *x*_3_^2^ coefficients), implying the existence of optimal ranges beyond which yield further diminishing returns or even reduced efficacy due to accelerated chlorine volatilization.

#### 3.2.4. Experimental Validation of the Model

To verify the predictive accuracy of the response surface models, eight additional validation experiments were conducted under randomly selected conditions within the optimized parameter ranges (Table 5). Each parameter combination was tested on both stainless steel and PVC surfaces, and the results were averaged across materials. The validation experiments covered cleaning times of 30–40 s, cleaning temperatures of 65–80 °C, and ACCs of 90–120 mg/L. As shown in Table 5, the observed values closely matched the model predictions. Linear regression analysis between observed and predicted values yielded *R*^2^ values of 0.968 for bacterial removal rate and 0.950 for ATP removal rate, demonstrating the strong predictive capability of both models. Paired *t*-tests further confirmed that there were no significant differences between the observed and predicted values for both bacterial removal rate (*p* = 0.308 > 0.05) and ATP removal rate (*p* = 0.975 > 0.05), verifying the high reliability of the established models.

### 3.3. Optimization of SAEW Cleaning Parameters

Based on the validated response surface models, optimal SAEW cleaning parameters were determined by maximizing both bacterial and ATP removal rates simultaneously. The optimal parameters identified were: cleaning time of 35 s (95% CI: 32–38 s, SE = 1.2 s), cleaning temperature of 78 °C (95% CI: 75–81 °C, SE = 1.1 °C), and an ACC of 108 mg/L (95% CI: 103–113 mg/L, SE = 2.5 mg/L), with a predicted bacterial removal rate of 100% and an ATP removal rate of 99.0%. Validation experiments conducted in triplicate on both stainless steel and PVC surfaces yielded a bacterial removal of 99.8 ± 0.2% (95% CI: 99.2–100.0%, SE = 0.3%) and an ATP removal of 98.7 ± 0.5% (95% CI: 98.1–99.9%, SE = 0.4%), both of which were within the 95% confidence intervals of the model predictions, confirming the accuracy and reliability of the RSM optimization.

SAEW cleaning parameters vary considerably depending on sanitation objectives, cleanliness requirements, and application methods. In poultry production, relatively low ACCs (50 mg/L) have been employed for disinfecting transport cages and vehicles [22,55], whereas microbial inactivation in layer hen houses typically require higher ACCs ranging from 80 to 250 mg/L [24,56,57]. For dairy farm milk handling systems, Liu et al. [27] reported optimal parameters of 9.9 min, 37.8 °C, and 60 mg/L ACC for stainless steel surfaces, and 14.4 min, 29.6 °C, and 60 mg/L ACC for rubber gaskets and PVC components, while Dev et al. [28] recommended 10 min, 39.8 °C, and pH 2.6 for acidic electrolyzed water treatment.

The optimal parameters identified in the present study are characterized by shorter cleaning durations combined with higher temperatures and ACCs. These differences reflect the specific operational constraints of AMF cleaning in calf rearing systems. First, the frequent feeding schedule of calves necessitates rapid cleaning cycles to minimize equipment downtime [42]. Second, milk residues combined with calf saliva may form more tenacious biofilms on feeding equipment surfaces compared to lactating cow milking systems, potentially requiring higher ACCs and temperatures to achieve effective sanitization.

### 3.4. Practical Considerations and Study Limitations

The relatively high ACC (108 mg/L) employed in this study warrants considerations of safety and economic implications. SAEW is non-toxic, non-corrosive at working concentrations, and decomposes rapidly into water and trace sodium chloride, leaving no harmful residues [58,59]. Previous studies have demonstrated that SAEW leaves minimal chemical residues compared to conventional chlorine-based sanitizers [60]. In calf feeding applications, the brief interval between cleaning and subsequent milk delivery provides sufficient time for residual chlorine dissipation. Nevertheless, routine monitoring of residual chlorine levels on equipment surfaces is recommended to ensure compliance with food-contact surface safety standards.

From an economic perspective, SAEW-based cleaning offers potential advantages over chemical sanitizers. SAEW can be generated on-site using only water, salt, and electricity, eliminating procurement and storage costs associated with commercial disinfectants [60]. The absence of harmful chemical residues reduces rinse water requirements and environmental compliance costs, while the relatively neutral pH of SAEW (pH 5.0–6.5) may cause less corrosion to equipment components compared with strongly alkaline or acidic cleaners, potentially extending equipment service life. Although the higher ACC and temperature identified in this study increase production and heating costs, this is partially offset by substantially reduced cleaning time, which decreases water consumption, labor costs, and equipment downtime. A comprehensive cost–benefit analysis incorporating direct costs (equipment, electricity, salt, water, labor) and indirect benefits (reduced calf morbidity, improved growth performance) would provide valuable guidance for farm-level decision-making.

Several limitations should be acknowledged when interpreting these results. First, the field study was limited to a single dairy farm and only two biological replicates, which may reduce the robustness and broader applicability of the findings. Second, the optimization experiments were conducted under controlled laboratory conditions using artificially contaminated specimens, which may not fully capture the complexity of mature biofilms on AMF surfaces in commercial farm settings. Additionally, the experimental runs in the RSM were not fully randomized, which may introduce potential run-order bias in the response values. Furthermore, this study only evaluated cleaning efficacy rather than direct effects on calf health. Future field trials with increased replication are warranted to verify protocol performance under practical conditions and to explore the relationship between improved AMF hygiene and calf health and growth.

## 4. Conclusions

This study confirmed that bacterial loads on five high-frequency calf-contact surfaces of uncleaned AMFs exceeded the widely accepted hygienic threshold for calf rearing equipment. Using RSM, we developed an optimized SAEW sanitation protocol, with parameters of 35 s cleaning time, 78 °C temperature, and 108 mg/L ACC. Under laboratory conditions, this protocol achieved a nearly 99% removal rate for both bacteria and ATP, with cleaning efficacy outperforming conventional chemical disinfectants. The short cleaning time of this optimized protocol minimizes equipment downtime and disruption to regular calf feeding. Overall, this work provides foundational data for SAEW application in AMF hygiene management, establishes SAEW as a sustainable alternative for rapid, effective cleaning of livestock equipment, and holds potential to improve calf health and welfare in group-housed systems in future applications.

## Figures and Tables

**Table 1:** Table of parameter design for single-factor experiments.

Factor	Test Parameter				
Time (s)	10	20	30	40	50
Temperature (°C)	20	35	50	65	80
ACC (mg/L)	30	60	90	120	150

**Table 2:** Box–Behnken response surface experiment parameter design table.

Group	Time (s)	Temperature (°C)	ACC (mg/L)
1	35	80	120
2	30	65	105
3	35	65	120
4	40	72.5	120
5	35	72.5	105
6	40	72.5	90
7	40	65	105
8	35	65	90
9	30	80	105
10	35	80	90
11	35	72.5	105
12	30	72.5	120
13	35	72.5	105
14	30	72.5	90
15	40	80	105
Chemical disinfectant	35	75	/

**Table 3:** Bacterial and ATP removal rates under different SAEW parameter combinations and control treatments. *n* = 3 for each group.

Solution	Group	Bacterial Removal Rate (%)		ATP Removal Rate (%)	
		PVC	Stainless Steel	PVC	Stainless Steel
SAEW	1	100.0 ± 0.2 ^1^	100.0 ± 0.2 ^1^	99.8 ± 0.5	99.6 ± 0.4
	2	85.8 ± 0.4	88.6 ± 0.3	85.0 ± 0.4	87.4 ± 0.6
	3	91.1 ± 0.1	91.5 ± 0.7	88.5 ± 0.9	90.9 ± 0.3
	4	100.0 ± 0.1 ^1^	100.0 ± 0.3 ^1^	100.0 ± 0.2 ^1^	100.0 ± 0.1 ^1^
	5	98.2 ± 0.2	97.4 ± 0.6	97.7 ± 0.6	95.5 ± 0.1
	6	94.3 ± 0.0	95.3 ± 0.3	95.0 ± 0.1	96.0 ± 0.4
	7	88.8 ± 0.5	90.4 ± 0.8	90.0 ± 0.6	89.8 ± 0.4
	8	81.1 ± 0.4	84.5 ± 0.5	80.5 ± 0.6	83.7 ± 0.5
	9	97.9 ± 0.6	97.5 ± 0.2	97.9 ± 0.3	97.6 ± 0.4
	10	97.5 ± 0.7	96.7 ± 0.4	95.8 ± 0.5	96.2 ± 0.2
	11	97.8 ± 0.2	97.1 ± 0.3	95.2 ± 0.3	97.4 ± 0.4
	12	97.3 ± 0.4	96.5 ± 0.6	97.2 ± 0.8	96.8 ± 0.6
	13	98.5 ± 0.2	97.8 ± 0.5	95.8 ± 0.2	98.0 ± 0.6
	14	88.1 ± 0.4	89.9 ± 0.7	89.2 ± 0.6	88.5 ± 0.3
	15	100.0 ± 0.2 ^1^	100.0 ± 0.1 ^1^	100.0 ± 0.2 ^1^	100.0 ± 0.1 ^1^
Chemical disinfectant		76.0 ± 0.7		72.0 ± 0.6	
No cleaning		0	0	0	0

^1^: “100%” simply indicates that contamination reduced to below detectable levels under the assay conditions, rather than implying absolute elimination. The same below.

**Table 4:** Analysis of variance for response surface model of sample plate bacterial removal rate and ATP removal rate.

Parameter	Bacterial Removal Rate		ATP Removal Rate	
	*F*-Value	*p*-Value	*F*-Value	*p*-Value
	64.06	0.000	85.40	0.000
*x*_1_	33.58	0.002	55.75	0.000
*x*_2_	349.37	0.000	481.90	0.001
*x*_3_	108.58	0.000	130.53	0.000
*x*_1_^2^	4.26	0.094	0.13	0.738
*x*_2_^2^	57.51	0.001	75.39	0.000
*x*_3_^2^	16.09	0.010	13.93	0.014
*x*_1_ *x*_2_	0.00	0.961	1.05	0.353
*x*_1_ *x*_3_	2.75	0.158	5.92	0.059
*x*_2_ *x*_3_	11.36	0.020	7.10	0.045
*R* ^2^	99.14%	/	99.35%	/
*R*^2^ (adjusted)	97.59%	/	98.19%	/
Lack of fit	/	0.094	/	0.101

where *x*_1_ is the cleaning time in s, *x*_2_ is the cleaning temperature in °C, and *x*_3_ is the ACC in mg/L.

**Table 5:** Trials with different parameters used to validate the models.

Time (s)	Temperature (°C)	ACC (mg/L)	Bacterial Removal Rate (%)		ATP Removal Rate (%)	
			Observed Value	Predicted Value	Observed Value	Predicted Value
30	65	90	78.0	79.5	81.4	78.8
30	75	120	96.5	98.2	96.1	98.2
30	80	110	98.5	98.0	99.9	98.0
35	65	100	85.7	87.3	83.4	85.8
35	80	110	100.0 ^1^	100.0 ^1^	99.5	99.1
40	70	90	93.9	91.8	91.6	92.4
40	75	110	100.0 ^1^	100.0 ^1^	100.0 ^1^	100.0 ^1^
40	80	95	97.2	99.3	99.1	98.9

Note: Observed values represent the mean of measurements on stainless steel and PVC surfaces (*n* = 3). Paired *t*-tests confirmed no significant differences between observed and predicted values for both response variables (*p* > 0.05). ^1^: “100%” simply indicates that contamination reduced to below detectable levels under the assay conditions, rather than implying absolute elimination.

## Data Availability

The data presented in this study are available on request from the corresponding author.

## Supplementary Materials

The following supporting information can be downloaded at: https://www.mdpi.com/article/10.3390/ani16081225/s1, Table S1: Comparison of bacterial and ATP removal rates between PVC and stainless steel surfaces across all RSM experiments.

## Abbreviations

The following abbreviations are used in this manuscript:

ACC	Available chlorine concentration
AMF	Automated milk feeder
AMFs	Automated milk feeders
APC	Aerobic plate count
ATP	Adenosine triphosphate
BPW	Buffered peptone water
CCE	Comprehensive cleaning efficiency
ClO^−^	Hypochlorite ions
CIP	Clean-in-place
R^2^	Coefficient of determination
HClO	Hypochlorous acid
LMM	Linear mixed model
RSM	Response surface methodology
PCA	Plate count agar
PVC	Polyvinyl chloride
SAEW	Slightly acidic electrolyzed water
TVC	Total viable count
