# Correction: Liu et al. Optimization of Sanitation Process Parameters of Slightly Acidic Electrolyzed Water for Automated Milk Feeders Using Response Surface Methodology. *Animals* 2026, *16*, 1225

**DOI:** 10.3390/ani16142217

**Published:** 2026-07-17

**Authors:** Yunying Liu, Yu Zhang, Xinyi Du, Zhengxiang Shi, Chaoyuan Wang, Hao Li, Amingguli Yasheng

**Affiliations:** 1 College of Water Resources and Civil Engineering, China Agricultural University, Beijing 100083, China; liuyunying@cau.edu.cn (Y.L.);; 2 Key Laboratory of Agricultural Engineering in Structure and Environment, Ministry of Agriculture and Rural Affairs, Beijing 100083, China; 3 Institute of Animal Husbandry and Veterinary Medicine, Anhui Academy of Agricultural Sciences, Hefei 230031, China; 4 Beijing Engineering Research Center on Animal Healthy Environment, Beijing 100083, China; 5 Beijing Honneur Agriculture and Technology Ltd., Beijing 101101, China

## Figure Legend

In the original publication [1], there was a mistake in the legend for Figure 2. Schematic diagram of the sampling template and paired sampling strategy. The Note of Figure 2 contains incorrect letter labels due to a writing error. The correct legend appears below.

**Figure 2.** Schematic diagram of the sampling template and paired sampling strategy. Note: Window A: Pre-cleaning swab sampling location for Pair 1; Window B: Post-cleaning swab sampling location (adjacent to D) for Pair 2; Window C: Post-cleaning swab sampling location (adjacent to A) for Pair 1; Window D: Pre-cleaning swab sampling location (adjacent to B) for Pair 2.

## Text Correction

1. There was an error in the original publication. A correction has been made to the Simple Summary: due to a typographical error, the unit log_10_ CFU/cm^2^ was incorrectly stated, and should be revised to log_10_ CFU/m^2^. The original sentence “We found bacterial loads on the feeder’s baffle, fence, and teat ranged from 5.48 to 8.21 log_10_ CFU/cm^2^, far exceeding the hygienic standard for calf feeding equipment”.

Detailed explanation:

Nature of the error: This is purely a unit labeling typo. All raw experimental data, dilution calculations, area conversion processes, and final numerical results are completely accurate; no recalculation of original data is required, and the scientific conclusions of the study remain entirely unchanged.

Rationale for reporting in log_10_ CFU/m^2^: As described in Section 2.2.3, the sterile sampling template defines a 3 × 3 cm (9 cm^2^) micro-area, and the surface area parameter S in Equation (1) is input in cm^2^ for the initial calculation of bacterial density. To maintain consistency with widely cited veterinary hygiene benchmarks and facilitate cross-study comparison, all final bacterial load results were uniformly converted to CFU per square meter (CFU/m^2^) for reporting, following the standard conversion: 1 m^2^ = 10,000 cm^2^. All unit labels throughout Section 3.1 of the main text are correctly stated as log_10_ CFU/m^2^, and the cm^2^ notation only appeared erroneously in the Simple Summary and Abstract during manuscript drafting.

Scope of correction: Only the unit notation in this section and the section below is revised; no other content is altered.

A corrected Simple Summary appears below:

Automated milk feeders (AMFs) are widely used on dairy farms to help calves feed naturally while reducing manual labor. However, if the feeders are not cleaned properly, harmful bacteria can build up on the surfaces that calves touch, leading to calf illness. This study quantified bacterial loads on key surfaces of feeders, compared slightly acidic electrolyzed water (SAEW) with chemical disinfectants, and optimized sanitation process parameters via response surface methodology. We found bacterial loads on the feeder’s baffle, fence, and teat ranged from 5.48 to 8.21 log_10_ CFU/m^2^, far exceeding the hygienic standard for calf feeding equipment. We identified optimal parameters: 35 s of cleaning time, 78 °C cleaning temperature, and 108 mg/L of available chlorine concentration, which removed 98% of bacteria. With optimal sanitation parameters, SAEW is effective and compatible with AMF applications. Our findings provide a technical reference for SAEW as a promising alternative sanitation agent for AMFs, offer a basis for standardized hygiene management of calf feeding equipment, and hold potential to support more sustainable dairy farming practices.

2. There was an error in the original publication. A correction has been made to the Abstract: due to a typographical error, the unit log_10_ CFU/cm^2^ was incorrectly stated, and should be revised to log_10_ CFU/m^2^. The original sentence “Results revealed that bacterial loads on five high-frequency calf-contact surfaces ranged from 5.48 to 8.21 log_10_ CFU/cm^2^”. The nature, rationale, and scope of this error are identical to those described in Item 1 above.

A corrected Abstract appears below:

Automated milk feeders (AMFs) offer significant advantages in promoting natural feeding behavior in calves and reducing manual labor. With widespread use, the impact of AMF hygiene on calf health has attracted increasing research attention, as inadequate cleaning protocols may lead to bacterial accumulation on calf-contact surfaces and subsequent health risks for calves. This study aimed to quantify bacterial contamination on AMF surfaces, evaluate the cleaning efficacy of slightly acidic electrolyzed water (SAEW) compared to warm water and chemical disinfectants (*n* = two total samples), and optimize SAEW cleaning parameters using response surface methodology (RSM). Results revealed that bacterial loads on five high-frequency calf-contact surfaces ranged from 5.48 to 8.21 log_10_ CFU/m^2^. SAEW at 60 mg/L achieved significantly higher cleaning efficacy than warm water and chemical disinfectants under field conditions (*p* < 0.01). Through RSM optimization (highly significant (*p* < 0.001)), the optimal SAEW cleaning parameters were determined as follows: cleaning time of 35 s, cleaning temperature of 78 °C, and available chlorine concentration (ACC) of 108 mg/L. Under the optimized parameters, bacterial and adenosine triphosphate (ATP) removal rates reached approximately 98%. These findings suggest that SAEW is a promising alternative sanitation agent for AMFs, provide preliminary parameters for rapid sanitation under the tested conditions, and hold the potential to support the standardized hygiene control of calf feeding equipment.

3. There was an error in the original publication. The letter labels describing the paired sampling strategy were incorrectly written due to a typographical error in Section 2.2.3 Cleaning Treatments. The original sentence was “Pair 1: pre-cleaning swab sampling from window A; following application of the assigned cleaning treatment, post-cleaning swab sampling from the adjacent window D. Pair 2: pre-cleaning swab sampling from window B; following application of the same treatment, post-cleaning swab sampling from the adjacent window C”.

A correction has been made to Section 2.2.3 Cleaning Treatments, Paragraph 2:

A paired sampling strategy was designed to eliminate the influence of spatial heterogeneity of surface contamination on cleaning efficacy evaluation. At each sampling site, a sterile portable template defining a 3 × 3 cm (9 cm^2^) micro-area was used for standardized sampling. The template contained four equal, non-overlapping square windows (Figure 2A–D). Two independent pre- and post-cleaning paired samples were collected for each treatment. Pair 1: pre-cleaning swab sampling from window A; following application of the assigned cleaning treatment, post-cleaning swab sampling from the adjacent window C. Pair 2: pre-cleaning swab sampling from window D; following application of the same treatment, post-cleaning swab sampling from the adjacent window B. All windows were positioned within the same micro-area to ensure baseline contamination homogeneity, while the non-overlapping design completely avoided cross-contamination between pre- and post-cleaning sampling. For S4, a flexible split-ring silicone template with two 1.5 × 1.5 cm windows was used. During the entire sampling and cleaning process, a dedicated handler kept calves away from the test area for approximately 10–15 min, and normal calf access to the pen was restored immediately after all operations were completed. All templates and disposable nitrile gloves were changed between sites to prevent cross-contamination.

4. There was an error in the original publication. Due to a typographical error, the term log_10_ was omitted in Section 3.1.1 Baseline Contamination Levels on AMF Surfaces. The original sentence “Böhm [31] proposed a general hygiene target of ≤3 CFU/cm^2^”.

A correction has been made to Section 3.1.1 Baseline Contamination Levels on AMF Surfaces:

Prior to cleaning, total viable count (TVC) ranged from 5.58 to 8.21 log_10_ CFU/m^2^ across the five sampling sites. Although no specific regulatory threshold exists for acceptable bacterial loads on AMF surfaces, Böhm [31] proposed a general hygiene target of ≤3 log_10_ CFU/cm^2^ (equivalent to ≤4.48 log_10_ CFU/m^2^) for surfaces in contact with animals following prophylactic sanitation. The high baseline bacterial counts observed in this study (5.58 to 8.21 log_10_ CFU/m^2^) clearly indicate substantial microbial contamination that far exceeds this acceptable hygiene standard. These findings underscore the critical importance of implementing regular and effective cleaning protocols for AMFs to minimize pathogen exposure and protect calf health.

5. There were some errors in the original publication. Due to a typographical error, the chemical formulas HClO and ClO^−^ were incorrectly spelled as HCIO and CIO^−^ throughout the published article, appearing in Section 1 Introduction, Paragraph 3, Section 3.1.2. Comparative Efficacy of Different Cleaning Agents, Paragraph 3, Section 3.2.2. Model Fitting and Adequacy Evaluation, Paragraph 5 and Abbreviations.

6. There was an error in the original publication. Due to a typographical error, in Section 2.5 Response Surface Modeling and Optimization, an extra asterisk was incorrectly added to the variable $x_{i}$ in the explanation of Equation (5), making it mistakenly written as $x_{i}^{*}$. The original sentence was “$x_{i}^{*}$ is the i-th independent variable”.

A correction has been made to Section 2.5 Response Surface Modeling and Optimization, Paragraph 2:

“where *CCE* is the comprehensive cleaning efficiency, serving as the unified optimization objective; $R_{ba}$ is the predicted bacterial removal rate (%) from the established response surface model; $R_{ATP}$ is the predicted ATP removal rate (%) from the established response surface model; $x_{i}$ is the i-th independent variable; $\Omega_{i}$ is the set experimental range of the i-th independent variable.”

7. There was an error in the original publication. Due to a typographical error, in Section 3.2.2 Model Fitting and Adequacy Evaluation, Paragraph 3, the linear coefficients (*x*_1_, *x*_2_, *x*_3_) were mistakenly written as (*x*_1_, *x*_2_, *x*_2_). The original sentence was “Analysis of individual model terms revealed that all linear coefficients (*x*_1_, *x*_2_, *x*_2_)”.

A correction has been made to Section 3.2.2 Model Fitting and Adequacy Evaluation, Paragraph 3:

“Analysis of individual model terms revealed that all linear coefficients (*x*_1_, *x*_2_, *x*_3_), the quadratic terms *x*_2_^2^ and *x*_3_^2^, and the interaction term *x*_2_*x*_3_ were significant (*p* < 0.05), whereas *x*_1_^2^, *x*_1_*x*_2_, and *x*_1_*x*_3_ were non-significant (*p* > 0.05) (Table 4). Based on *F*-values, the relative importance of factors followed the order: cleaning temperature > ACC > cleaning time for both response variables.”

The authors state that the scientific conclusions are unaffected. This correction was approved by the Academic Editor. The original publication has also been updated.
