# Supplementary material for: Optimization of Sanitation Process Parameters of Slightly Acidic Electrolyzed Water for Automated Milk Feeders Using Response Surface Methodology
Source: Animals (Basel). 2026 Apr 16;16(8):1225. doi: 10.3390/ani16081225 (PMC13113120; doi:10.3390/ani16081225)
Supplement: Supplementary file 1 [file animals-16-01225-s001.zip › animals-4196962-supplementary.pdf]

**Table S1. Comparison of bacterial and ATP removal rates between PVC and stainless steel surfaces across all RSM experiments.** Values are means ± standard error (SE)

| Material | Bacterial removal rate (%) | ATP removal rate (%) |
|---|---|---|
| PVC | 94.4 ± 0.9 | 93.8 ± 0.9 |
| Stainless steel | 94.9 ± 0.7 | 94.5 ± 0.8 |
| *P*-value | P=0.337 | P=0.166 |
